# Criteria for Verification and Replanning Based on the Adaptive Radiotherapy Protocol “Best for Adaptive Radiotherapy” in Head and Neck Cancer

**DOI:** 10.3390/life12050722

**Published:** 2022-05-12

**Authors:** Bartosz Bak, Agnieszka Skrobala, Anna Adamska, Joanna Kazmierska, Natalia Jozefacka, Tomasz Piotrowski, Julian Malicki

**Affiliations:** 1 Department of Electroradiology, Poznan University of Medical Science, 61-866 Poznan, Poland; agnieszka.skrobala@wco.pl (A.S.); joanna.kazmierska@wco.pl (J.K.); tomasz.piotrowski@wco.pl (T.P.); julian.malicki@wco.pl (J.M.); 2 Department of Radiotherapy II, Greater Poland Cancer Centre, 61-866 Poznan, Poland; 3 Department of Medical Physics, Greater Poland Cancer Centre, 61-866 Poznan, Poland; 4 Department and Radiotherapy Ward I, Greater Poland Cancer Centre, 61-866 Poznan, Poland; anna.adamska@wco.pl; 5 Institute of Psychology, Pedagogical University in Krakow, 30-084 Krakow, Poland; natalia.jozefacka@up.krakow.pl

**Keywords:** radiotherapy planning, replanning, adaptive radiotherapy, head and neck cancer, image-guided radiation therapy, helical tomotherapy

## Abstract

No clear criteria have yet been established to guide decision-making for patient selection and the optimal timing of adaptive radiotherapy (ART) based on image-guided radiotherapy (IGRT). We have developed a novel protocol—the Best for Adaptive Radiotherapy (B-ART) protocol—to guide patient selection for ART. The aim of the present study is to describe this protocol, to evaluate its validity in patients with head and neck (HN) cancer, and to identify the anatomical and clinical predictors of the need for replanning. We retrospectively evaluated 82 patients with HN cancer who underwent helical tomotherapy (HT) and subsequently required replanning due to soft tissue changes upon daily MVCT. Under the proposed criteria, patients with anatomical changes >3 mm on three to four consecutive scans are candidates for ART. We compared the volumes on the initial CT scan (iCT) and the replanning CT (rCT) scan for the clinical target volumes (CTV1, referring to primary tumor or tumor bed and CTV2, metastatic lymph nodes) and for the parotid glands (PG) and body contour (B-body). The patients were stratified by primary tumor localization, clinical stage, and treatment scheme. The main reasons for replanning were: (1) a planning target volume (PTV) outside the body contour (*n* = 70; 85.4%), (2) PG shrinkage (*n* = 69; 84.1%), (3) B-body deviations (*n* = 69; 84.1%), and (4) setup deviations (*n* = 40; 48.8%). The replanning decision was made, on average, during the fourth week of treatment (*n* = 47; 57.3%). The mean reductions in the size of the right and left PG volumes were 6.31 cc (20.9%) and 5.98 cc (20.5%), respectively (*p* < 0.001). The reduction in PG volume was ≥30% in 30 patients (36.6%). The volume reduction in all of the anatomical structures was statistically significant. Four variables—advanced stage disease (T3–T4), chemoradiation, increased weight loss, and oropharyngeal localization—were significantly associated with the need for ART. The B-ART protocol provides clear criteria to eliminate random errors, and to allow for an early response to relevant changes in target volumes.

## 1. Introduction

Most patients who undergo radiotherapy for head and neck (HN) cancer experience anatomical alterations during treatment, mainly due to the shrinkage of the primary tumor and/or metastatic lymph nodes, changes in body contours due to weight loss, or a decrease in parotid gland volumes. These changes present a significant challenge because they can lead to inappropriate dose distribution in the target area, with the potential for uncontrolled dose escalation to the organs at risk (OAR). Adaptive radiotherapy (ART), which is now considered the “state-of-art” of modern radiotherapy, was developed in order to minimize the effects of these changes. In clinical practice, ART is mainly based on image-guided radiotherapy (IGRT), which allows clinicians to verify patient positioning, and to visualize ongoing anatomical changes. As the term “adaptive radiotherapy” suggests, the original treatment plan must be adapted in order to address these anatomical changes, which is achieved through replanning. Numerous studies have shown that these radiation-induced anatomical changes mainly occur during the first phase of the radiotherapy course [1,2,3,4,5,6,7,8]. The decision to modify the original treatment plan depends on several key variables, mainly changes in body weight (primarily weight loss) and changes in the volume and position of the target area. In patients who meet the criteria for replanning, ART has several important advantages, including a lower risk of late adverse effects and the potential to improve disease-free survival [9,10,11,12,13,14].

Advanced treatment modalities such as volumetric arc therapy (VMAT) and helical tomotherapy (HT) permit modifications to the original treatment plan at all stages of treatment. A survey on the use of ART in Europe involving 177 institutions in 40 different countries found that only 10% of public centers used online or offline protocols for HN cancer; of these, only 28% made ad-hoc offline adaptations [15]. In addition, the survey found that most adaptations were performed in order to improve target coverage while sparing OARs. The primary imaging modality for ART is either cone-beam CT (CBCT) or mega-voltage CT (MVCT) [15,16].

Despite the growing importance of ART, a recent literature review concluded that there is a notable lack of standardization in the selection criteria used to determine the need for replanning [17]; this finding is consistent with most of the studies conducted to date, which have concluded that clear selection criteria are needed in order to accurately determine the indication for ART [1,2,3,18,19]. At present, patient selection is based on images obtained through computed tomography (CT) and/or IGRT scans performed either weekly or randomly. To our knowledge, no specific imaging criteria are currently available to guide patient selection.

In this context, we developed a novel protocol at our institution, which we called the “Best for Adaptive Radiotherapy” (B-ART) protocol. We created this protocol to provide objective criteria to establish patient eligibility for ART. To our knowledge, this is the first study to propose clear guidelines based on IGRT for the indication for ART in HN cancer. We believe that this protocol could significantly reduce radiotherapy department workloads while also potentially improving treatment outcomes.

The present study has three main aims: (1) to describe the B-ART protocol and its underlying assumptions, (2) to assess its validity for HN cancer radiotherapy and (3) to identify anatomical and clinical predictors for replanning decision-making. We compared volume changes during the course of radiotherapy in the primary tumor (CTV1), lymph nodes (CTV2), parotid glands (PG), and body contour (B-Body) between the initial pretreatment values and those obtained during replanning. We also evaluated a set of key variables—age, gender, primary tumor site, TNM clinical stage, and treatment regimen (radiotherapy alone or chemoradiotherapy)—in order to determine the factors most closely correlated with the need for replanning.

## 2. Materials and Methods

### 2.1. Patient Demographic and Clinical Characteristics

This was a retrospective analysis of 82 patients with newly-diagnosed, biopsy-proven HN squamous cell carcinoma (HNSCC) radically treated with helical tomotherapy (HT) (Accuray Inc., Sunnyvale, CA, USA). Most of the patients (*n* = 63; 76.8%) underwent concurrent chemotherapy (CHT). Table 1 shows the demographic and clinical characteristics of the sample. The most common cancer types were oropharyngeal (OPC; 45%), oral cavity (OCC; 20%), and laryngeal (15%) cancer. Most of the patients had advanced-stage disease (stage T4, 62%; and N2, 61%). Of the 63 patients who received CHT, 43 (68.2%) were prescribed weekly cisplatin (40 mg/m^2^) and 18 (28.6%) received 100 mg/m^2^ cisplatin every three weeks. Two patients (3.2%) received weekly cetuximab. *n* = 26 (32%) patients underwent post-operative treatment (PORT).

### 2.2. Treatment Planning and Replanning

All of the patients were planned and treated on HT, and were immobilized with a five-point custom-fitted thermoplastic face and shoulder mask (Civco Medical Solutions, Kalona, IA, USA). The initial computed tomography (iCT) imaging for planning purposes was performed on a Siemens Definition AS scanner using 3-mm slice spacing. CT data were loaded into the Aria—Eclipse v. 13.6 software (Varian, Palo Alto, CA, USA). The gross tumor volume (GTV) was defined as the pathological mass visible on iCT images fused with intravenous contrast CT. The clinical target volume (CTV1) was defined as the GTV plus a margin to account for potential microscopic spread. CTV2 was defined as the high-risk nodal region. The CTVs were expanded by 3 mm to create the planning target volumes (PTV). All of the OARs were contoured according to the DAHANCA EORTC consensus guidelines [20]. The body contour structure covered the area from the apex of the skull to the sternum (suprasternal notch) at the level of the T3 vertebrae.

The treatment plans were optimized to ensure that 95% of the PTV received 100% of the prescribed dose, with ≤2% of the PTV volume receiving 107% of the dose. The total doses to the primary tumor ranged from 60 to 70 Gy. The median dose fraction was 2 Gy (range 1.8–2 Gy). The dose schedule in the 82 patients was as follows: 70 Gy/35 fractions (fx]) (*n* = 64; 78%), 66 Gy/33 fx (*n* = 10; 12%), 60 Gy/30 fx (*n* = 6; 7%), and 61.2 Gy/34 fx (*n* = 2; 2%).

New replanning CT scans were acquired for all 82 patients, and were used to replan the HT treatment plan for the remaining treatment fractions. The treating radiation oncologist (RO) attempted to maintain the original CTVs to the greatest extent possible, making only the modifications required to adapt the plan to the specific changes detected in the anatomical structures.

### 2.3. The B-ART Protocol

During the course of radiotherapy, the patients underwent daily MVCT scans and weekly physical examinations. According to the B-ART protocol, the daily MVCT scan area includes the GTV and adjacent critical organs (which are considered sufficient to assess the patient’s anatomy) in order to detect potential emerging alterations and to correct patient positioning. The value of limiting the daily scanning area to the GTV alone is that it reduces the verification time and imaging-related radiation doses for the patients.

Image verification was performed once weekly for the entire PTV and all of the OARs included in the planning process [21,22]. The anatomical image visualized on the MVCT was compared to the iCT using an image fusion algorithm in the HT system. The B-ART protocol criteria used to determine the patient eligibility for ART are shown in Table 2. The assessment of accuracy was based on the reconstruction of the bone structure and soft tissue. Changes >3 mm at any point of the external body contour on three to four consecutive scans were considered significant (B-ART criteria).

**Figure 1:**
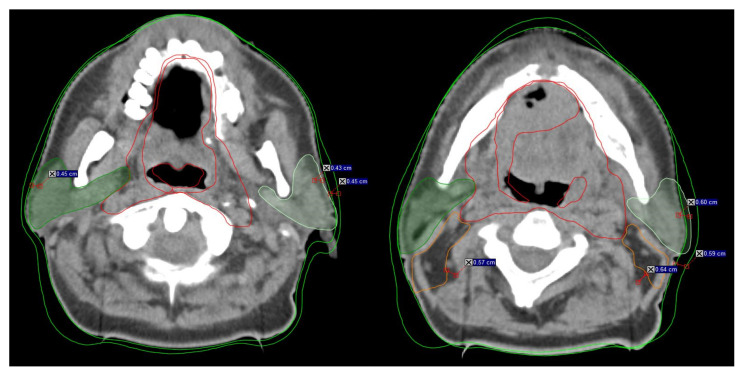
Example of the rapidly changing anatomy during head neck radiotherapy from treatment initiation to replanning. Visible changes >3 mm in the anatomy during RT indicate inclusion for B-ART. The distance mark on the scans represents the anatomy changes between initial CT (iCT) and replanning (rCT). Dark green indicates the right parotid gland, and light green indicates the left parotid gland. Red indicates CTV1, orange indicated CTV2, and green indicates the external contour (B-body).

The initial image assessment was the responsibility of the radiation therapists (RTT) in accordance with the daily B-ART protocol, which stipulates that all shifts >3 mm be reviewed (Figure 1). The RO was then informed about any issues regarding OAR and target volume coverage. After consultation with the RTT, the RO then made the replanning decision. The reasons for replanning were documented, with the most common reasons being: (1) PTV outside the body, (2) PG shrinkage, (3) body contour alterations, and (4) setup deviations. The eligibility criteria for ART were based on a detailed evaluation of the CTVs (CTV1, CTV2), PGs, and body contour, especially volumetric changes upon daily imaging. Special attention was paid to the interrelation between those structures and soft tissues, muscles, and other OARs. Under the protocol, the RTTs were instructed to ensure that all of the following criteria were met: (1) the CTV contour should include no more than 10% of the PG volume, (2) metastatic lymph nodes (CTV2) in nodal levels II–IV must not involve the sternocleidomastoid muscle or levator scapulae, (3) the PTV should not extend beyond the body contour, and (4) PGs should not shift medially in the high-dose region (Table 2). If any of these four points were violated, the patients were referred for replanning, and rCT scans were acquired. In order to reduce inter-observer variability in the contouring, the same RO who prepared the initial plan also performed the recontouring.

For the present study, we compared differences in the volumes between the initial and replanning CTs (ΔV[iCT/rCT]) for the primary tumor (CTV1), metastatic lymph nodes (CTV2), right and left parotid glands, and body contour. Both the absolute (ΔVcc) and percentage (ΔV%) volume reductions were estimated (Table 3). In order to identify potential predictors (anatomical and/or clinical) of the need for replanning, we evaluated the correlation between ΔV and the following variables: age, sex, radiotherapy alone (yes/no), concomitant chemotherapy (yes/no), postoperative radiotherapy (PORT, yes/no), and primary tumor location (nasopharynx, oropharynx, hypopharynx, larynx, other). Finally, we assessed the time at which the replanning was performed.

### 2.4. Statistical Analysis

The correlation of the volume reduction (VR) as ΔV(cc) and ΔV(%) between (iCT/rCT) parameters was assessed by means of the Kruskall–Wallis, Spearman’s, *t*-test or Wilcoxon test. The patients were stratified by subgroup according to the cancer diagnosis (OPC, OCC, and “other”), and then compared.

Spearman’s test was used to determine the correlation between the volume differences between the iCT and rCT images and age. For the variables for sex and clinical diagnosis, the differences between the iCT and rCT images were tested using non-parametric tests (the Mann–Whitney U test and Kruskal–Wallis test, respectively).

The correlation between the volume reduction (ΔVcc and ΔV%) and staging (TNM) in all of the groups, stratified by diagnostic subgroups (OPC/OCC/other), was based on parametric ANOVA for normally distributed data and the non-parametric Kruskal-Wallis test for non-normal distributions. The Bonferroni test for differences was performed, with a cut-off value of α = 0.05 for statistical significance. The IBM-SPSS Statistical software package (v.26, Chicago, IL, USA) was used to perform the statistical analyses.

## 3. Results

The main reasons given by the RTTs to prequalify patients for ART in this series were as follows: (1) PTV located outside the body (*n* = 70; 85.4%), (2) PG shrinkage or repositioning (*n* = 69; 84.1%), (3) weight loss resulting in visible changes in body contour (*n* = 69; 84.1%), and (4) setup deviations, defined as difficulties in patient positioning and/or adjustments to the initial plan (*n* = 40; 48.8%). These results from IGRT verification and the percentage differences by diagnosis, staging, and treatment scheme are given in Table 3. All 82 patients (100%) recommended for ART by the RTTs according to the B-ART criteria were approved for replanning by the RO. Table 4 shows the anatomical changes based on the B-ART protocol. There were no statistically significant correlations between CTV1 VR and any of the following variables: age, gender, primary tumor site, and treatment scheme (RT/CHT/PORT).

Compared to the baseline, the mean tumor (CTV1) volume reduction was 11.67 cc (range, 7.61–15.73), which is a 5.31% decrease (*p* = 0.001). The changes in the CTV1 were larger in patients with more advanced disease: stages T4 vs. T1 (*p* = 0.001); T4 vs. T2 (*p* = 0.044); and T3 vs. T1 (*p* = 0.007). The patients with stage T3/T4 disease had greater weight loss (represented by their B-body volume reduction) and more anatomical alternations in the clinical target area.

The mean CTV2 (nodal) volume reduction was 7.73 cc (range, 4.73–10.66 cc), an 8.4% decrease (*p* < 0.001). There was a statistically significant difference between the volume reduction in the CTV2 and the clinical stages N1 (*p* = 0.027), N2 (*p* < 0.001), and N3 (*p* = 0.049), but not for stage N0 (*p* = 0.202). The CTV2 volume differences were more significant in patients with stage T4 tumors (mean, 14.20 cc; *p* = 0.024).

The maximum absolute and percent volume reductions for the left and right PGs were 5.98 cc (range, 4.76–7.20; 20.5% volume reduction) and 6.31 cc (5.16–7.45; 20.9%), respectively (*p* < 0.001). Table 5 shows the mean and percentage volume changes in the PGs, stratified by diagnosis. After a mean of 20 fractions, volume reductions ≥30% were observed in 36.6% of the right PGs (*n* = 30) and 34.1% of the left PGs (*n* = 28) (Table 6). Nine patients (11%) experienced an increase in volumes in the right PG, and 13 patients (15.9%) experienced this in the left.

### Replanning Decision

Table 3 summarizes the four major discrepancies detected during image guidance that suggested a potential need for replanning. The replanning decision was made upon the detection of any of the aforementioned anatomical changes on MVCT, most commonly 3–4 mm contour shifts on three consecutive scans (Table 2).

In this cohort, ART was initiated, on average, at the 20th fraction (range: 6–29 fx, standard deviation (SD), 5.3). In most patients (*n* = 47; 57.3%) replanning was performed at the fourth week (Table S1). In most cases (*n* = 77; 94%) the replanning decision was made during the first phase of treatment, even though the adapted plan was not administered until the second phase of treatment (i.e., after the fifth week) in most cases (*n* = 51, 68.3%) due to the time required to create a new plan (mean, 3 days).

## 4. Discussion

Currently, the selection criteria for treatment replanning in patients with HN cancer remain poorly defined. As a result, patient selection for ART is usually based on weekly or random CT scans and/or on the clinical judgement of the treating radiation oncologist. The results of this preliminary study, based on four years of data, demonstrate that the B-ART model can accurately determine the optimal time to perform ART based on daily imaging scans obtained by IGRT.

Careful analysis of the anatomical changes evidenced on IGRT-based imaging can potentially help us to predict subsequent changes in the primary tumor, lymph nodes, parotid glands, and body volume during radiotherapy. The B-ART protocol can therefore help us to identify the patients who most likely to benefit from replanning. According to the protocol criteria, if the difference between the patient’s external (body) contour on the MVCT and the iCT is greater than 3 mm on at least three to four scans (3-mm slices) on any of the treatment volumes (CTV, PTV, or PG), the RTT must notify the radiation oncologist, who must then confirm that the patient meets the protocol criteria and schedule a new CT image for replanning (Table 2, Figure 1).

The simple assumption underlying this protocol—i.e., that anatomical changes on three consecutive scans are a significant finding—may eliminate random errors and improve decision-making. Importantly, we found that the initial recommendation for ART by the RTT (based on the protocol criteria) was confirmed by the RO in 100% of cases, thereby underscoring the value of the B-ART protocol and the proposed replanning criteria (Table 3). Given the current lack of standard replanning protocols, there is an undeniable need for clear protocols to guide decision-making in these cases. Several authors have emphasized this need, including Brown et al. [1], who found that only 15 of 110 patients in their study were included for replanning. We believe that our protocol includes the most comprehensive set of selection criteria to date. The time at which replanning becomes necessary varies widely in HN cancers. Similarly, the probability that replanning will be necessary depends on many variables. Our data show that the factors most closely associated with replanning are large nodal volumes, concurrent chemotherapy, increased weight loss, oropharyngeal localization, and an advanced primary tumor. Table S2 describes in detail the risk profile for ART.

In the published literature, rescanning is generally performed for four main reasons: (1) IGRT verification [1,2,3,10,23,24] (2) weekly treatment control [CT imaging] [1,2,3,4,5,6,13,18,23,24,25,26,27,28], (3) visible changes in anatomy (weight loss, thermoplastic mask mismatch) [2,3,7,8,10,18,23,24], and (4) cumulative dose calculations based on CBCT or MVCT scans [3,23,29,30,31], which we described and summarized in a previous study [17]. Despite the lack of guidelines to determine patient eligibility for replanning, weekly follow-up CT scans may be helpful. Unfortunately, this approach significantly increases the departmental workload, and also involves additional, unnecessary radiation.

In our series, the most common time at which replanning was considered necessary was around fraction 20, which corresponds to the fourth week of treatment (Table S1). However, the adapted treatment was started between fractions 21 and 23 (week 5 of treatment), mainly due to the time needed to prepare the new plan. Our findings regarding the timing of ART are consistent with previous reports, even though those studies used conventional methods (random or weekly CT scans) to determine the need for replanning [1,3,4,7,10,17,24,28,32]. These findings support the validity and value of the B-ART protocol: in our experience, the application of this protocol has two main benefits: (1) a substantial reduction in departmental workload, and (2) decreased patient exposure to unnecessary radiation doses from KV imaging.

Anatomical changes in patients with HN often start well before they become visually evident, which is why daily IGRT is essential. Tumor shrinkage, weight loss, and changes in the parotid glands are three common reasons for performing ART. When indicated, replanning is vital; otherwise, there is a high risk of inappropriate target irradiation and OAR overdosing [7,10,33,34]. In a study of 31 HNSCC patients who underwent replanning (10.6% of the sample), Figen et al. [34] found that the most common reasons for replanning were tumor shrinkage (35.5%) and weight loss (35.5%). In addition, in line with our findings, most of the patients (62%) had stage IV disease. Many studies have found that a reduction in tumor size, which is often detected within the first two weeks of treatment, is an indicator that replanning is necessary. In the literature, the median shrinkage rates range from 16–66% after 2–7 weeks of treatment [1,4,13,17,18,26,27,30,31,32,33,35,36]. In fact, a decrease in the size of the primary tumor is the main predictor for ART. Zhao et al. [36] evaluated 175 patients with stage II–IV (AJCC criteria) nasopharyngeal cancer (NPC) treated with IMRT, observing anatomical changes in 158 patients (90.3%). In the replanned patients, the mean volume reduction of the primary tumor after 20 fractions was 10.75 cc (±32.51), with a mean nodal volume reduction of 17.27 cc (±22.46).

In our study, the mean CTV1 volume reduction was 11.67 cc (5.31%). In more advanced tumors (stage T3/T4), we observed greater changes in the CTV1 volume. Similarly, those patients also experienced more weight loss and greater anatomical alterations in the clinical target area compared to the patients with smaller tumors. The mean CTV2 (nodal) volume reduction was 7.7 cc (8.4%), with significant differences between the volume reduction in stage N3 (*p* = 0.049) and T4 tumors (*p* = 0.001).

It is worth noting that the volume changes in our study were, in general, smaller than those reported in the literature. This finding further supports the validity of the B-ART protocol, as statistically significant changes in volumes were detected earlier than in other studies, allowing us to perform the replanning as soon as possible.

We found body volume changes in most of the patients (84.1%), especially during chemoradiation (83%) (Table 3). We also found that body alterations (mainly B-body volume reduction) were closely correlated with changes in lymph node volumes (CTV2) in nodal levels II–IV. Moreover, our data indicate that changes >3 mm on three to four consecutive scans in the nodal region would have led to inappropriate dose distributions to the OARs and CTV; similarly, in most cases, the PTV exceeded the body’s external contour, which is associated with an increased risk of more adverse effects and/or undertreatment. In the majority (85.4%) of our replanned patients (Table 3), ART was indicated because the PTV contour was outside the body (Table 2). Interestingly, we found that body contour changes occurred nearly simultaneously with salivary gland shrinkage, which affected the position of the lymph nodes in levels II–IV. The mean nodal volume reduction was greater in the replanned patients diagnosed with OCC compared to OPC or other cancer types, but this difference was not statistically significant.

In general, displacements >3 mm in a specific anatomical area of the PGs (the actual contour in relation to the body’s initial external contour or surrounding muscles) were considered significant. The midline shifting of the PGs was detected in 84.1% of the patients. Yao et al. [3] observed weekly changes in the PG from the first to the 33rd fraction. In that study, replanning was considered necessary if the cumulative parotid dose was >10% of the initial planning dose. Those authors found that the parotid volume variation rate peaked at the 16th fraction and then gradually decreased, suggesting that the fourth week is the optimal time for replanning. Vásquez et al. [37] evaluated 10 patients with OPC, reporting volume loss in the PGs, with a medial shift of 3 mm and a change in the mean planned dose to both glands. The dosimetric consequences of PG shrinkage vary widely, with a mean PG dose increase of 2.2 ± 2.7 Gy [1,3,4,5,6,7,25,26,32,35]; in some studies, the overdose can be as high as 4 Gy [1,8,13,18,19,31,33,38,39].

During a typical course of radiotherapy in patients with HN cancer, the average volume reduction in the PGs is 26% [3,4,6,8,11,13,18,21,24,26,32,40,41]. We grouped the PG volume changes into five groups according to the percentage change (Table 6). Most of the patients (*n* = 30; 36.6%) experienced a volume reduction ≥30%. The greatest absolute and percentage reductions in PG volume were 6.31 cc (20.9%) and 5.98 cc (20.5%) for the right and left PGs, respectively. These results are consistent with the findings obtained by other researchers [10,13,26]. In patients with OPC, the largest percentage volume reductions in the right and left iPG were 27% (7.2 cc) and 25% (6.0 cc), respectively (Figure 1), indicating that >39% of these patients would require replanning, and should therefore be included in the high-risk group. By contrast, Brown et al., estimated that only 2% of OPC patients versus up to 67% of node-positive NPC patients (stage N3) would require replanning [1], leading them to conclude that some patients with other HN tumors (e.g., OPC) may be considered high risk depending on the tumor characteristics. However, given the small number of patients who underwent ART in that study, those findings should be interpreted cautiously. Nevertheless, as our data show, ART has a profound impact on HN patients in general, not only on high-risk groups such as those with OPC or NPC with advanced nodal disease. Our findings indicate that replanning during IMRT is essential for all HN patients undergoing CRT; this same conclusion applies to the PORT group, although the results were not quite as strong.

The B-ART protocol is a novel selection protocol for ART based on image verification. To date, only four other groups [2,28,39,40] have proposed specific criteria for online anatomical changes. In most cases, the authors of those studies recommended performing another CT if the difference between the planning CT and the CBCT/MVCT was >1 cm at any point on the external contour within the treatment area; nevertheless, specific anatomical changes were often poorly described, without any clear guidelines. By contrast, in the excellent study by Duma et al. [2], the authors proposed a key criterion for replanning: the presence of soft tissue changes > 0.5 cm on one side. In that study, if only minimal (e.g., 2–4 mm) soft tissue changes were detected, then changes in the delivered doses were not considered relevant. However, if the initial plan resulted in delivered doses close to the tolerance doses, especially when image guidance was not performed daily, then the dose decrease could be significant.

In most countries, the replanning decision is based on one of two approaches, both of which rely on imaging verification (IGRT). In the first approach, the images provide a sufficient foundation for decision-making and the re-optimization of treatment plans. A second—less common and more expensive—approach (and thus not feasible for routine use in many centers) involves the use of dedicated tools to calculate the actual dose distribution in the OARs and target area based on the cumulative dose from CBCT or MVCT, or by the application of deformable image registration algorithms such as Velocity, MIM, or Ray Station.

According to the results of the ESTRO-HERO study, the staffing levels in Europe meet or exceed the QUARTS (Radiation Therapy for Cancer: Quantification of Radiation Therapy Infrastructure and Staffing Needs) recommendations [42]. However, that study also found substantial variability among countries in terms of staffing levels, and that human resource needs have increased as modern RT techniques—such as real-time respiratory motion management (RRMM) and ART—have become more complex. In this context, replanning is time consuming, which is why software is essential in order to help reduce the workload. In the survey conducted by Bertholet et al. [15], half of the respondents who reported using only in-house software for ART in patients with HN and lung cancer stated that cost was the main reason they did not use commercial software. Fifty-seven users ranked the barriers to expanding or modifying their ART technique for an existing tumor site, with the main barriers being human/material resources and technical limitations. Over half of respondents performed ad-hoc adaption, but less than a third used specific protocols. In general, CBCT was the main imaging modality, but some users also reported using magnetic resonance for daily replanning [15,16].

## 5. Conclusions

The B-ART protocol—based primarily on the anatomical analysis of structures such as the PG, CTV, and PTV—has the potential to become the “gold standard” in the determination of patient eligibility for replanning. This protocol relies on a simple, clear concept to indicate the need for ART: changes >3 mm on 3–4 consecutive scans at any point of the external contour are considered significant. In our series, all 82 patients who met this criterion were approved for replanning by the radiation oncologist, and underwent ART. Compared to the initial treatment plan, all of the anatomical structures in all of the patients showed a significant volume reduction, which clearly indicates that replanning was necessary, in most cases during the fourth week of treatment.

Our main aims in developing the B-ART protocol were to decrease patient exposure to unnecessary radiation doses from random re-scanning, and to reduce the departmental workload. In all of the HN patients undergoing IMRT, it is essential to monitor volume changes in key structures, and to perform replanning if needed. Close observation is especially important in patients with advanced tumors, those undergoing combined chemoradiotherapy, patients who exhibit substantial weight loss, and those with oropharyngeal cancers. Our data show that patients with these characteristics are more likely to require ART, and should therefore be considered high risk.

We believe that the proposed protocol will be particularly relevant and of interest to centers that need general guidelines for replanning patients with HN cancers, especially those that do not have access to external systems for ad-hoc plan deformation and recalculation.

## Figures and Tables

**Table 1:** Demographic and clinical characteristics of the 82-patient cohort, and their treatment details.

Patient Characteristics and Treatment Details			
Characteristic		Value (Range)	(%)
Age		59 years (23–83)	
Sex			
	F	17	21%
	M	65	79%
Diagnosis			
	OPC	37	45%
	OCC	16	20%
	L	12	15%
	MS	7	9%
	HPC	4	5%
	NPC	3	4%
	CUP	2	2%
	NCC	1	1%
Stage (TNM)			
	T1	8	10%
	T2	7	9%
	T3	16	20%
	T4	51	62%
	N0	11	13%
	N1	17	21%
	N2	50	61%
	N3	4	5%
Radiotherapy (RT) scheme			
	RT (alone)	19	23%
	PORT	26	32%
	CRT	63	77%

Abbreviations: OPC = oropharyngeal cancer; NPC = nasopharyngeal cancer; OCC = oral cavity cancer; L = laryngeal cancer; HPC = hypopharyngeal cancer; CUP = cancer of unknown primary; NCC = nasal cavity cancer; MS = maxillary sinus; CRT = chemoradiotherapy; RT = radiotherapy; PORT = postoperative RT.

**Table 2:** B-ART protocol selection criteria for ART.

Structure	Alert	Assessment	Criteria
Parotid Glands	The difference between the iCT and the CBCT/MVCT at any point of the external contour	Superficial part of PG (the area near masseter muscle)	>3 mm on 3–4 consecutive scans
	PG shrinkage	Deep part of the PG lobe	
	PG shift medially in the high-risk region	Medial pterygoid muscle	
CTV 1 (Tumor)	The difference between the iCT and the CBCT/MVCT in any direction	CTV1 contour and position changes	>3 mm on 3–4 consecutive scans
	CTV1 overlaps OARs (muscles, PG, bones, air cavities)		
CTV 2 (Nodal region)	The difference between the iCT and the CBCT/MVCT, especially in the nodal levels II–IV	CTV2 contour and position changes	>3 mm on 3–4 consecutive scans
	CTV2 overlaps OARs (muscles, PG, bones, air cavities)		
PTV	The difference between the iCT and the CBCT/MVCT at any point of the external contour (PTV outside the body)	PTV contour and position changes	>3 mm on 3–4 consecutive scans
		Body contour/weight changes	

Abbreviations: CBCT, cone-beam computed tomography; MVCT, mega-voltage computed tomography; iCT, initial computed tomography; PG, parotid gland; OAR, organ at risk; CTV, clinical target volume; PTV, planning target volume; ART, adaptive radiotherapy.

**Table 3:** The B-ART protocol: decision on ART based on IGRT observation and selection criteria; PTV outside the body; PG shrinkage +/− reposition; weight loss, visible B-body contour changes; setup deviations, IGRT matching.

		PTV		Parotid Glands		Weight Loss		Setup Deviations	
		(Outside the Body)							
		N/% of Total							
No		12	14.6 %	13	15.9 %	13	15.9 %	42	51.2 %
Yes		70	85.4 %	69	84.1 %	69	84.1 %	40	48.8 %
		**PTV**		**Parotid Glands**		**Weight Loss**		**Setup deviation**	
**Diagnosis**	N (%)	Yes	No	Yes	No	Yes	No	Yes	No
CUP	2 (2%)	2 (100%)	_	2 (100%)	_	2 (100%)	_	1 (50%)	1 (50%)
HPC	4 (5%)	4 (100%)	_	3 (75%)	1 (25%)	4 (100%)	_	2 (50%)	2 (50%)
L	12 (15%)	9 (75%)	3 (25%)	9 (75%)	3 (25%)	10 (83%)	2 (17%)	5 (42%)	7 (58%)
MS	7 (9%)	6 (86%)	1 (14%)	6 (86%)	1 (14%)	5 (71%)	2 (29%)	6 (86%)	1 (14%)
NCC	1 (2%)	_	1 (100%)	1 (100%)	_	_	1 (100%)	1 (100%)	_
NPC	3 (5%)	3 (100%)	_	2 (67%)	1 (33%)	3 (100%)	_	2 (67%)	1 (33%)
OCC	16 (20%)	13 (81%)	3 (19%)	13 (81%)	3 (19%)	15 (94%)	1 (6%)	6 (38%)	10 (63%)
OPC	37 (45%)	33 (89%)	4 (11%)	33 (89%)	4 (11%)	30 (81%)	7 (19%)	17 (46%)	20 (54%)
**T-Stage**									
T1	8 (10%)	7 (88%)	1 (13%)	6 (75%)	2 (25%)	7 (88%)	1 (13%)	5 (63%)	3 (38%)
T2	7 (9%)	6 (86%)	1 (14%)	6 (86%)	1 (14%)	5 (71%)	2 (29%)	2 (29%)	5 (71%)
T3	16 (20%)	14 (88%)	2 (13%)	14 (88%)	2 (13%)	15 (94%)	1 (6%)	6 (38%)	10 (63%)
T4	51 (62%)	43 (84%)	8 (16%)	43 (84%)	8 (16%)	42 (82%)	9 (18%)	27 (53%)	24 (47%)
**N-Stage**									
N0	11 (13%)	8 (73%)	3 (27%)	10 (91%)	1 (9%)	9 (82%)	2 (18%)	5 (45%)	6 (55%)
N1	17 (21%)	12 (71%)	5 (29%)	16 (94%)	1 (6%)	12 (71%)	5 (29%)	6 (35%)	11 (65%)
N2	50 (61%)	47 (94%)	3 (6%)	39 (78%)	11 (22%)	45 (90%)	5 (10%)	26 (52%)	24 (29%)
N3	4 (5%)	3 (75%)	1 (25%)	4 (100%)	_	3 (75%)	1 (25%)	3 (75%)	1 (25%)
		**PTV**		**Parotid glands**		**Weight Loss**		**Setup deviation**	
**Radiotherapy**		Yes	No	Yes	No	Yes	No	Yes	No
No	63 (77%)	54 (86%)	9 (14%)	52 (83%)	11 (17%)	52 (83%)	11 (17%)	32 (51%)	31 (49%)
Yes	19 (23%)	16 (84%)	3 (16%)	17 (89%)	2 (11%)	17 (89%)	2 (11%)	8 (42%)	11 (58%)
**Radiochemotherapy**									
No	19 (23%)	16 (84%)	3 (16%)	17 (89%)	2 (11%)	17 (89%)	2 (11%)	8 (42%)	11 (58%)
Yes	63 (77%)	54 (86%)	9 (14%)	52 (83%)	11 (17%)	52 (83%)	11 (17%)	32 (51%)	31 (49%)
**Postoperative radiotherapy**									
No	53 (65%)	49 (92%)	4 (8%)	48 (91%)	8 (15%)	47 (89%)	9 (17%)	26 (49%)	30 (57%)
Yes	26 (32%)	21 (81%)	5 (19%)	21 (81%)	5 (19%)	22 (85%)	4 (15%)	14 (54%)	12 (46%)

Abbreviations: PTV, planning target volume; B-body, contour changes; IGRT, image-guided radiotherapy; OPC, oropharyngeal cancers; OCC, oral cavity cancers; NPC, nasopharynx; L, larynx; HPC, hypopharynx; CUP, cancer of unknown primary; NCC, nasal cavity cancer; MS, maxillary sinus cancer.

**Table 4:** Mean absolute (cc) and percentage (%) volume changes in the anatomy in the patient sample (*n* = 82).

Anatomy Structure		iCT Only (Range)	SD	rCT Replan (Range)	SD	*p*-Value *
CTV1		iCTV1		rCTV1		**<0.001**
Mean		150.5 cc	90.9	138.8 cc	82.6	
Median (range)		136.9 (6.8–494.8) cc		124.9 (6.7–449) cc		
Mean CTV1 volume regression ΔV				−11.67 cc (5.3%)	18.6	
CTV2		iCTV2		rCTV2		**<0.001**
Mean		88.1 cc	12.9	80.4 cc	15.5	
Median (range)		59.25 (10.8–403.9) cc		54.20 (9–379.9) cc		
Mean CTV2 volume regression ΔV				−7.73 cc (8.4%)	12.9	
GTV		iGTV		rGTV		**<0.001**
Mean		55.1 cc	45.4	49.6 cc	42.7	
Median (range)		39.20 (2.6–192.2) cc		36.50 (1.2–177.2) cc		
Mean percentage GTV volume regression				−5.26 cc (9.5%)	7.05	
Parotid gland [Right]		iPG [R]		rPG [R]		**<0.001**
Mean		24.7 cc	8.7	18.4 cc	6.1	
Median (range)		24.6 (5.3–50.4) cc		17.8 (3.4–35.6) cc		
Mean percentage PG[R] volume regression				−6.31 cc (20.9%)	6.3	
Parotid gland [Left]		iPG [L]		rPG [L]		**<0.001**
Mean		23.9 cc	8.9	17.9 cc	4.1	
Median (range)		22.6 (7.2–42.5) cc		16.8 (4.1–34.5) cc		
Mean percentage PG[L] volume regression				−5.98 cc (20.5%)	6.3	

Abbreviations: iCTV, initial clinical target volume; rCTV, replanning target volume; cc, cubic centimeter; ΔV, volume difference between the initial and replanning CT; HN, head and neck; iGTV, initial gross tumor volume; rGTV, replanning gross tumor volume; iPG, initial parotid gland; rPG, replanning parotid gland; SD, standard deviation. * A *p*-value of α = 0.05 was considered statistically significant.

**Table 5:** Mean and percentage volume changes in the parotid glands, stratified by diagnosis. The initial diagnosis was grouped according to the highest representation in the analyzed cases.

	Diagnosis	N	Mean	Median	SD	Range	
Parotid gland—iPG [Right]	OPC	37	24.9 cc	25.6 cc	8.3	5.3	39.6
	OCC	16	25.8 cc	24.5 cc	9.2	9.8	50.4
	Other	29	23.8 cc	22.5 cc	9.3	9.4	48.9
Parotid gland—rPG [Right]	OPC	37	17 cc	16.9 cc	5.3	3.4	31.2
	OCC	16	19.2 cc	17.6 cc	7.1	9.5	35.6
	Other	29	19.6 cc	19.5 cc	6.4	9.9	33.5
Parotid gland [Right] Mean difference	OPC	37	−7.2 cc	−7.4 cc	7.2	−23.7	14.8
	OCC	16	−5.2 cc	−6.7 cc	6.1	−15	6.9
	Other	29	−4.2 cc	−3.3 cc	5.1	−18.1	5.7
Parotid gland [Right] Percentage difference	OPC	37	−27%	−31%	22.1	−77%	45%
	OCC	16	−19%	−24%	20.9	−48%	28%
	Other	29	−14%	−18%	20.1	−48%	45%
Parotid gland [Left]—iPG	OPC	37	23.7 cc	22.8 cc	8.4	7.2	42.5
	OCC	16	24.0 cc	21.8 cc	8.3	13.1	39.8
	Other	29	24.2 cc	22.2 cc	10.1	8.5	42.2
Parotid gland [Left]—rPG	OPC	37	16.6 cc	15.7 cc	6.0	4.1	31.2
	OCC	16	19.6 cc	18.8 cc	6.5	10.4	32.4
	Other	29	18.8 cc	17.3 cc	7.3	6.3	34.5
Parotid gland [Left] Mean difference	OPC	37	−6.0 cc	−6.0 cc	7.0	−27.7	17.2
	OCC	16	−4.1 cc	−3.4 cc	5.0	−13.1	3.6
	Other	29	−5.4 cc	−5.1 cc	6.1	−16.3	3.9
Parotid gland [Left] Percentage difference	OPC	37	−25%	−25%	21.7	−72%	48%
	OCC	16	−15%	−16%	18.5	−44%	17%
	Other	29	−18%	−19%	24.8	−55%	31%

Abbreviations: iPG, initial parotid glands; rPG, replanning parotid glands; OPC, oropharyngeal cancers; OCC, oral cavity cancers; and Other (NPC, nasopharynx; L, larynx; HPC, hypopharynx; CUP, cancer of unknown primary; NCC, nasal cavity cancer; MS, maxillary sinus cancer).

**Table 6:** Categorization of the parotid glands according to the percentage volume changes (ΔV%) in all 82 HN patients, stratified by cancer localization.

	PG [Right]		PG [Left]		PG [Right]						PG [Left]					
Diagnosis	All Group of Patient				OPC		OCC		Other		OPC		OCC		Other	
	N	%	N	%	N	%	N	%	N	%	N	%	N	%	N	%
Increase volume	9	11%	13	15.9%	3	8%	2	13%	4	14%	6	21%	3	19%	4	11%
Volume reduction ≤5%	5	6.1%	6	7.3%	1	3%	2	13%	2	7%	2	7%	2	13%	2	5%
Volume reduction [5–10%]	8	9.8%	3	3.7%	3	8%	_	_	5	17%	2	7%	_	_	1	3%
Volume reduction [10–20%]	14	17.1%	15	18.3%	4	11%	3	19%	7	24%	6	21%	4	25%	5	14%
Volume reduction [20–30%]	16	19.5%	17	20.7%	7	19%	3	19%	6	21%	3	10%	4	25%	10	27%
Volume reduction ≥30%	30	36.6%	28	34.1%	19	51%	6	38%	5	17%	10	35%	3	19%	15	41%

Abbreviations: HN, head and neck; PG, parotid gland; N, number of patients; OPC, oropharyngeal cancers; OCC, oral cavity cancers; and Other (NPC, nasopharynx; L, larynx; HPC, hypopharynx; CUP, cancer of unknown primary; NCC, nasal cavity cancer; MS, maxillary sinus cancer).

## Data Availability

The datasets analyzed in the current study are available from the corresponding author upon request.

## Supplementary Materials

The following supporting information can be downloaded at: https://www.mdpi.com/article/10.3390/life12050722/s1. Table S1. Replanning decision timing. Table S2. Adaptive radiotherapy risk profile.
