# Supplementary material for: Criteria for Verification and Replanning Based on the Adaptive Radiotherapy Protocol “Best for Adaptive Radiotherapy” in Head and Neck Cancer
Source: Life (Basel). 2022 May 12;12(5):722. doi: 10.3390/life12050722 (PMC9144703; doi:10.3390/life12050722)
Supplement: Supplementary file 1 [file life-12-00722-s001.zip › Table S1. Replanning decision timing.pdf]

Table S1. Replanning decision timing

Decision of replanning by phase of treatment			Initiation of replanning by phase of treatment		
Phase	N	% of Total	Phase	N	% of Total
1	77	93.9 %	1	26	31.7 %
2	5	6.1 %	2	51	62.2 %
3	-	-	3	5	6.1 %

Frequency of replanning decision (N-x fraction)			Frequency of fraction from (replan to start)		
N-x fraction of ART decision	Counts	% of Total	N-x fraction of new start	Counts	% of Total
6	4	4.9 %	10	5	6.1 %
7	1	1.2 %	14	2	2.4 %
9	2	2.4 %	16	2	2.4 %
11	2	2.4 %	17	2	2.4 %
12	2	2.4 %	19	2	2.4 %
14	1	1.2 %	20	2	2.4 %
15	1	1.2 %	21	3	3.7 %
16	3	3.7 %	22	1	1.2 %
17	4	4.9 %	26	55	67.1 %
19	3	3.7 %	27	1	1.2 %
20	7	8.5 %	31	4	4.9 %
21	12	14.6 %	6	1	1.2 %
22	13	15.9 %	8	1	1.2 %
23	10	12.2 %	15	1	1.2 %
24	5	6.1 %			
25	7	8.5 %			
26	1	1.2 %			
27	2	2.4 %			
29	2	2.4 %			

Frequencies of ART replanning Decision - week			Frequencies of ART replanning decision week from replan to start		
Week of replanning decision	Counts	% of Total	Week of new plan	Counts	% Of Total
2	7	8.5 %	2	7	8.5 %
3	6	7.3 %	3	11	13.4 %
4	17	20.7 %	4	4	4.9 %
5	53	64.6 %	5	56	68.3 %
6	5	6.1 %	6	4	4.9 %
