# Supplementary material for: Criteria for Verification and Replanning Based on the Adaptive Radiotherapy Protocol “Best for Adaptive Radiotherapy” in Head and Neck Cancer
Source: Life (Basel). 2022 May 12;12(5):722. doi: 10.3390/life12050722 (PMC9144703; doi:10.3390/life12050722)
Supplement: Supplementary file 1 [file life-12-00722-s001.zip › Table S2. Adaptive Radiotherapy Risk Profile.pdf]

Table S2. Adaptive Radiotherapy Risk Profile

Adaptive strategy – Risk Profile			
	**Low risk**	**Intermediate risk**	**High risk**
Tumor localization	NCC/MS//HPC	**NPC/OPC/L**	***NPC/OPC/OCC/L***
Loose mask		*Loose mask detected in weeks 3-4*	*Loose mask detected during week 1*
BMI/B-body	*If initial weight <100 kg*	*If initial weight <100 kg*	*Initial weight >100 kg*
CHRT		*no chemoradiation*	*chemoradiation*
Postoperative RT		*No*	*Yes*
Stage	***T1/N0***	***T2-T3 / <N1***	***T3-T4 / N1-N3***
Parotid Glands		*parotid glands also move to the body midline during radiation therapy*	*parotid glands also move to the body midline during radiation therapy*
		difference between the planning scan and the CBCT/MVCT are greater than 3mm on 3-4 consecutive scans at any point of the external contour	*difference between the planning scan and the CBCT/MVCT are greater than 3mm on 3-4 consecutive scans at any point of the external contour*
Nodal Status		*Dominant pretreatment nodal in region II-IV*	*Dominant pretreatment nodal in region II-IV*
			CTV moves outside the body during treatment
			CTV covering more than 10% of initial PG volume
PTV			*PTV outside the Body during treatment >3mm on 3-4 consecutive scans*
**Suggested control Replan-CT**	***Observation***	***2-5th week of treatment***	***3-5th week of treatment***

Abbreviations: H&N, head and neck; PG, parotid gland; CTV, clinical target volume; PTV, planning target volume; BMI, body mass index; CHRT, chemoradiotherapy; OPC, oropharyngeal cancer; OCC, oral cavity cancers; and Other (NPC, nasopharynx, L=larynx, HPC=hypopharynx, CUP=cancer of unknown primary, NCC=nasal cavity cancer, MS=maxillary sinus cancer).
